# Photoacoustic imaging for monitoring radiotherapy treatment response in head and neck tumors

**DOI:** 10.1038/s41598-025-95137-0

**Published:** 2025-05-10

**Authors:** Niklas Holzwarth, Zoe Rachel, Jan-Hinrich Nölke, Melanie Schellenberg, Lukas Bauer, Nicholas Schreck, Christoph J. Bender, Kris K. Dreher, Sebastian Regnery, Katharina Weusthof, Manuel Wiesenfarth, Annette Kopp-Schneider, Jürgen Debus, Alexander Seitel, Sebastian Adeberg, Lena Maier-Hein, Thomas Held

**Affiliations:** 1 Division of Intelligent Medical Systems (IMSY), German Cancer Research Center (DKFZ), Heidelberg, Germany; 2 Faculty of Mathematics and Computer Science, Heidelberg University, Heidelberg, Germany; 3 Department of Radiation Oncology, Heidelberg University Hospital, Heidelberg, Germany; 4 HIDSS4Health, Helmholtz Information and Data Science School for Health, Heidelberg, Germany; 5 National Center for Tumor Diseases (NCT), NCT Heidelberg, a Partnership Between DKFZ and University Medical Center Heidelberg, Heidelberg, Germany; 6 Biostatistics Division, German Cancer Research Center (DKFZ), Heidelberg, Germany; 7 Faculty of Liberal Arts and Sciences, Augsburg University of Applied Sciences, Heidelberg, Germany; 8 Faculty of Physics and Astronomy, Heidelberg University, Heidelberg, Germany; 9 Clinical Cooperation Unit Radiotherapy, German Cancer Research Center (DKFZ), Heidelberg, Germany; 10 Department of Radiotherapy and Radiation Oncology, Marburg University Hospital, Marburg, Germany; 11 Marburg Ion-Beam Therapy Center (MIT), Marburg University Hospital, Marburg, Germany; 12 University Cancer Center (UCT), Frankfurt-Marburg, Germany; 13 Medical Faculty, Heidelberg University, Heidelberg, Germany

**Keywords:** Head and neck cancer, Photoacoustic imaging, Radiotherapy, In vivo, Xerostomia, Head and neck cancer, Photoacoustics

## Abstract

Head and neck (HN) tumors are responsible for approximately 4% of annual new cancer cases worldwide. Besides surgery, radiochemotherapy, particularly fractionated radiotherapy (RT), is the gold-standard treatment modality for these cancers. However, there is currently no reliable early measure of success available to further personalize treatment plans. This work aims to address this critical bottleneck by pioneering the use of photoacoustic imaging (PAI) to measure treatment response in HN cancer patients undergoing RT. PAI leverages the photoacoustic effect in order to non-invasively recover functional tissue properties in depths of up to several centimeters. We hypothesized that oxygen saturation ($\text {sO}_2$), hemoglobin concentration, and water content, as measured by PAI, would non-invasively reflect expected RT treatment effects, namely reoxygenation of lymph nodes (hypothesis H1), inflammation of surrounding organs (H2) and xerostomia (H3). Our study with n = 30 human subjects showed notable changes in $\text {sO}_2$, hemoglobin concentration, and water levels in HN tumor patients resulting from disease treatment. Our data confirmed hypotheses H2 and H3, while an observed decrease in $\text {sO}_2$ over the treatment course contradicted our prior assumptions (H1). A comprehensive analysis based on device and tissue digital twins, however, revealed that low blood volume fraction as encountered in malignant nodes, can lead to particularly high $\text {sO}_2$ prediction errors, indicating that the measured $\text {sO}_2$ values cannot be trusted within these regions. We conclude that our study is the first to show that PAI is capable of measuring early molecular changes induced by RT in human tissue non-invasively. Further studies are now needed to convert the potential of the new imaging technique into patient benefit.

## Introduction

Squamous cell carcinoma represents the predominant subgroup of head and neck (HN) tumor malignancies^1^ with an annual occurrence of about 890,000 diagnoses representing about 4.5% of new cancer cases worldwide and resulting in around 450,000 fatalities annually (GLOBOCAN estimate)^2^. These tumors are characterized by large tumor volume and local invasion, usually with evidence of a high rate of metastasis to regional lymph nodes^3^. Besides surgery, primary radiochemotherapy is an equivalent treatment choice for locally advanced head and neck squamous cell cancer (LA-HNSCC) with corresponding 5-year overall survival rates of around 50%^4^ and locoregional control rates of approximately 60% at 2-years^5^. Individualized decision-making regarding initial therapy, sequencing, and administration requires expertise in balancing local tumor control, general patient morbidity, toxic effects of therapy, and functional preservation of treated structures. Currently, there is no established routine of clinical evaluation during radiotherapy (RT) aimed at assessing early treatment success, particularly with regard to molecular parameters. The first treatment response evaluation typically occurs 3 months after RT and involves examination by an ear, nose, and throat (ENT) specialist, along with imaging (magnetic resonance imaging (MRI) or computed tomography (CT))^6,7^. Therefore patients receive a CT scan to evaluate tumor response following the RECIST 1.1 criteria^8^, undergo a clinical examination, and report therapy-related side effects using the Common Terminology Criteria for Adverse Events (CTCAE)^9^ questionnaire.

**Fig. 1:**
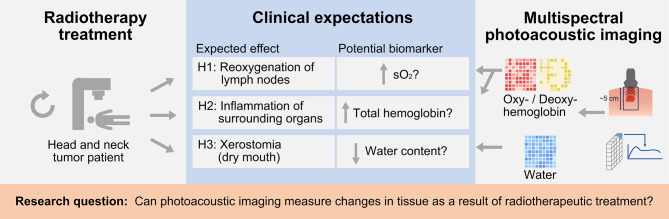
Mission of this work: to investigate whether photoacoustic imaging can measure changes in tissue caused by radiotherapeutic treatment. The specific focus was to analyze whether clinically expected short- (H1) and long-term (H2 and H3) effects resulting from radiotherapy manifest in photoacoustic measurements. This figure was created with Affinity Designer 2 (Version 2.5.3, https://affinity.serif.com/de/designer/). The photoacoustic probe subfigure was created with Adobe Illustrator (CC 2015, https://www.adobe.com/products/illustrator.html).

The main effects of RT occur on a molecular level, involving processes such as inflammation, revascularization, and reoxygenation. Molecular changes arise as a result of the interaction of ionizing radiation with tissues and cells, triggering a cascade of responses that may either resolve quickly (early molecular changes) or lead to long-term modifications (late molecular effects). Early effects emerge within seconds to hours after radiation exposure and involve processes primarily related to deoxyribonucleic acid (DNA) damage and the activation of cellular stress responses, such as reactive oxygen species (ROS) production, leading to earlier clinical manifestations such as mucositis and radiodermatitis in the HN area^10^. Late molecular changes can occur weeks to years after radiation exposure and result from persistent damage, chronic oxidative stress, and long-term cellular dysfunction. These effects often reflect the tissue’s inability to completely repair damage and adapt, for instance, endothelial dysfunction and vascular damage in which radiation-induced vascular injury triggers chronic inflammation and reduced perfusion. This, in the long-term, contributes to late fibrosis, organ dysfunction, or necrosis, as it is seen in patients after RT, who report persistent xerostomia. Various factors can influence the biological effects of radiotherapy in human tissues. For instance, the presence of oxygen enhances the radiotherapeutic effect by prolonging the lifespan of free radicals and reducing the cells’ ability to repair sublethal DNA damage^11,12^. Tumor tissue often exhibits intratumoral hypoxia, resulting in radioresistance, which can be addressed by fractionating radiotherapy to leverage reoxygenation effects. Hence, there’s a significant need for patient selection, particularly in identifying those who may not benefit from conventional RT and should receive adapted or alternative treatment approaches like dose escalation or salvage surgery, or those who exhibit deep responses and could be candidates for RT de-escalation to minimize side effects. This approach aligns with the concept of ”personalized medicine” as a fundamental principle in modern treatment planning^13^. One of the major side effects, salivary gland damage, stands as the prevailing long-term complication of both RT and chemoradiotherapy in HN cancer treatment. Xerostomia can result in notable symptoms like dry mouth and contribute to additional complications such as dental caries and nutritional challenges. Alterations in saliva quantity and composition shortly after starting radiation therapy indicate that these glands undergo acute and delayed responses^14^. There is no standard objective method to assess xerostomia^15^ and we lack imaging modalities that enable us to directly assess early treatment success beyond tumor shrinkage. A promising emerging opportunity in this context is photoacoustic imaging (PAI). PAI is an innovative biomedical imaging modality that utilizes a combination of laser light and ultrasound to image functional hemodynamic properties of tumors in vivo in a dose-free manner. PAI leverages the optical absorption properties of hemoglobin to generate estimates of total hemoglobin concentration (tHb) and oxygen saturation ($\text {sO}_2$). A major advantage of PAI is its ability to quantify these parameters without requiring exogenous contrast agents, making it a noninvasive technique. In contrast, conventional radiological methods such as MRI, positron emission tomography (PET), or CT often rely on external tracers. The utility of PAI is currently being investigated in various preclinical and clinical studies. Among others, it is being explored for the detection of thyroid carcinoma^16^, for photoacoustic mammography^17^, and for assessing inflammatory activity in Crohn’s disease^18^. Of particular interest in tumor-investigative studies is the characterization of tumor tissue and the tumor microenvironment^19^. Studies by Becker et al.^20^ demonstrated with PAI that reactive and metastatic lymph nodes exhibit differences in chromophore concentrations ex vivo, while Rich et al.^11^ showed in mice that PAI is capable of assessing head and neck tumor hemodynamics during RT. However, the potential benefit of PAI for RT response has not yet been investigated in patients. Given the gap in the literature, this study aims to explore the potential of PAI in assessing the response to RT in malignant lymph nodes of head and neck tumors in vivo. To achieve this objective, we conducted the first explorative patient study on head and neck tumor patients who underwent repeated imaging sessions throughout RT treatment with PAI. Our approach involved matching the expected treatment effects with potential biomarkers extractable using PAI (Fig. 1). Specifically, we hypothesized the following effects of RT:

1. An early molecular increase in oxygenation in lymph nodes resulting from treatment.
2. A late increase in total hemoglobin, indicative of inflammation.
3. A late decrease in saliva production and, consequently, reduced water content in salivary glands.

## Results

To investigate the three hypotheses, we conducted a longitudinal study involving 15 young healthy volunteers and 15 patients. We successfully included eleven patients with no sign of progress in disease after 3 months of follow-up (ten patients showed partial response and one patient was classified as stable disease following the RECIST criteria). These patients were scanned before, during (after the 9th RT fraction), and 90 days post-therapy using an MSOT Acuity Echo hybrid ultrasound and photoacoustic device. A detailed description of the study design can be found in the methods section.

### Measured oxygenation does not reflect prior knowledge

**Fig. 2:**
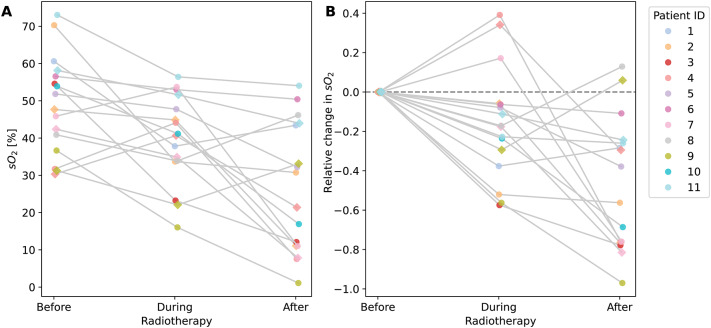
Oxygen saturation ($\text {sO}_2$) before, during (after the 9th fraction), and 90 days after radiotherapy (RT) treatment in suspect lymph nodes, as measured by photoacoustic imaging (PAI), decreases over time. This is shown for the overall $\text {sO}_2$ values (**A**) and relative to the pre-RT $\text {sO}_2$ (**B**), with each node color-coded by patient. For some patients, multiple nodes were measured, as indicated by their shape (1st node: circle; 2nd node: diamond shape).

According to the literature (e.g. Steel et al.^21^), we anticipated reoxygenation of malignant lymph nodes resulting from RT treatment (H1). In contrast to this expectation, our PAI measurements indicated a discernible decline in the estimation of the biomarker $\text {sO}_2$ within most of the malignant target lymph nodes as a result of RT (Fig. 2). In particular, we observed a strong increase in deoxyhemoglobin levels during and after RT, without a pronounced trend for oxygenated hemoglobin (Supplementary Figs. S1 and S2). For visual reference, images depicting a malignant lymph node throughout RT are presented in the Supplementary information Fig. S3.

### Linear mixed model analysis reveals largely unexplained signal variability

For better interpretability of our results, we performed an analysis of the explained variation using the variance component form of the linear mixed model (LMM)^22^. In an ideal imaging setting, most variability in measurement would be explained by therapy-induced differences in tissue structure and function such as reoxygenation, revascularization, or necrosis. However, real-life imaging is influenced by numerous additional factors contributing to the signal generation for example subject, depth of the organ of interest, and seasonal variations, potentially shadowing the actual target property. Our results indicate that the variance of $\text {sO}_2$ in our measurements is largely unexplained (40%). Only 32% is treatment-induced (temporal variation) while 32% can be attributed to intra-subject variability (Supplementary Table S1).

### Digital twin provides insight into unexpected oxygenation measurements

**Fig. 3:**
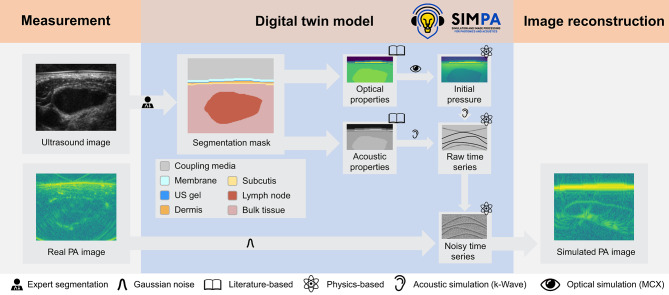
A comprehensive simulation pipeline enables the generation of tissue digital twins. To obtain synthesized images that are geometrically consistent with our measured data, ultrasound images (US) are first converted into a semantic representation (segmentation mask) by clinical experts. The segmentation masks are then annotated with plausible optical and acoustic tissue properties obtained from the literature. Finally, existing methods for photon (Monte Carlo eXtreme—MCX) and acoustic wave (k-Wave) propagation as well as realistic noise generation, provided by the SIMPA toolkit^23^, are applied to generate a digital twin photoacoustic (PA) image. This figure was created with Affinity Designer 2 (Version 2.5.3, https://affinity.serif.com/de/designer/https://affinity.serif.com/de/designer/).

**Fig. 4:**
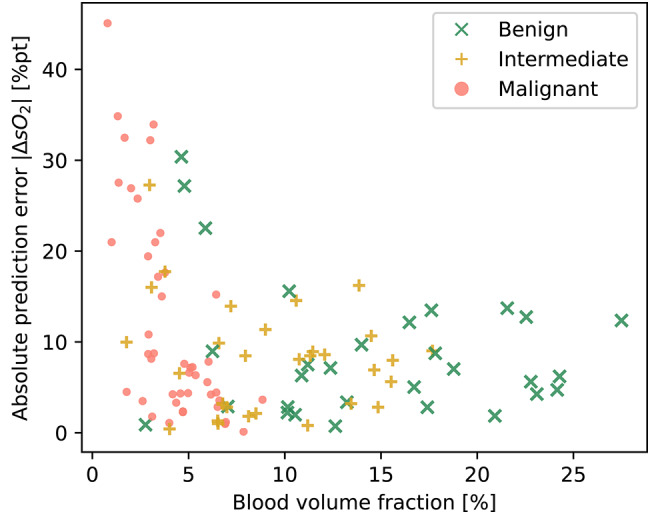
Digital twin analysis reveals a strong link between oxygen saturation ($\text {sO}_2$) prediction error (y-axis) and low blood volume fraction (x-axis) for benign, intermediate, and malignant lymph nodes. Every cross represents the mean absolute $\text {sO}_2$ error of one digital twin scan with the $\text {sO}_2$ ground truth defined by the assigned optical input properties.

A major issue in PAI, in general, is the lack of ground truth for the provided measurements, hindering the accurate assessment of biological effects. However, the hybrid US-PAI technology provides us with reliable information about tissue morphology. This morphological data, when coupled with literature-derived optical and acoustic properties, enables us to develop digital tissue twins, potentially circumventing the ground truth limitation. With this tissue twin model, we can simulate different biological effects with exact knowledge of the ground truth e.g. oxygenation. Leveraging the ground truth knowledge we can analyze how the biological change influences the photoacoustic device twin measurement. This method was used for a deeper analysis of the unexpected oxygenation measurements (decline in lymph node $\text {sO}_2$). To this end, expert physicians converted the measured data into semantic segmentation masks, resulting in a labeled dataset with four tissue classes (dermis, subcutis, bulk tissue, and lymph node) and three material classes (coupling media, membrane, and ultrasound gel), as illustrated in Fig. 3. These served as the basis for generating tissue digital twins, which, in turn, were used for the synthesis of PAI images annotated with ground truth physiological properties. For each tissue twin, prior knowledge of optical and acoustic tissue properties was leveraged to simulate three clinically informed^24^ disease states malignant, benign, and artificial intermediate (details see methods). The digital twin analysis revealed that low blood volume fraction (BVF), as encountered in malignant nodes, can lead to particularly high $\text {sO}_2$ prediction errors (Fig. 4). In other words, the measured $\text {sO}_2$ values cannot be trusted in regions of low BVF.

### Photoacoustic imaging suggests signs of inflammation in the sternocleidomastoid muscle after radiotherapy

**Fig. 5:**
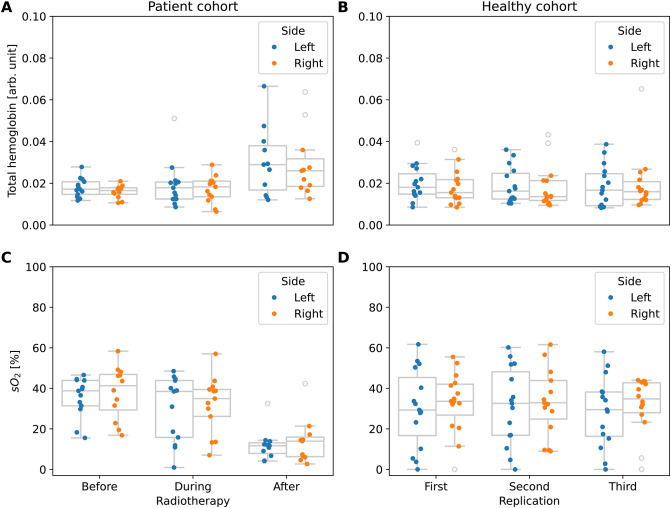
Photoacoustic imaging indicates evidence of inflammation in the sternocleidomastoid muscle following radiotherapy (RT) treatment, characterized by an increase in total hemoglobin (**A**). The increase is mainly caused by deoxyhemoglobin (Supplementary Fig. S4) leading to a drop in oxygen saturation ($\text {sO}_2$) (**C**). Healthy volunteer data (no RT—three temporal replications at one session) are displayed for comparison (**B**/**D**). The box shows the interquartile range (IQR) with the median as center line. The whiskers extend to points that lie within 1.5 * IQR of the 1st or 3rd quantile. All data points are plotted on top, and color-coded by measurement side (left or right neck). Outliers are shown without color.

Current RT treatments are becoming progressively more and more targeted. Nonetheless, a considerable portion of the radiation is still delivered in organs surrounding the primary target (here: lymph nodes) resulting in local inflammation^25^. In support of H2, we observed an increase in tHb concentration in the sternocleidomastoid muscle, especially at the last (third) measurement (Fig. 5). The tHb estimates before and during RT, on the other hand, are in a similar signal range to the measurements of the healthy cohort (Fig. 5B). The observed increase in tHb is mainly due to an increase in deoxyhemoglobin, which approximately doubled between the second and third measurements indicating tissue activity (Supplementary Fig. S4). This, coupled with a drop in oxygenated hemoglobin, results in a decline in derived $\text {sO}_2$.

It is a well-known fact that the functional tissue parameters provided by the PAI system can only serve as rough approximations of the ground truth, primarily due to the spectral coloring effect in tissue^26^. To overcome this issue, we investigated the tumor-to-background ratio, which is a common measure to derive radiopharmaceutical uptake using PET^27,28^. To differentiate between surrounding and tumor tissue response to radiation we calculated the tumor-to-muscle (TMR) ratio for $\text {sO}_2$. This ratio exhibits an increasing trend (Supplementary Fig. S5), primarily driven by a strong increase in deoxyhemoglobin under RT of the sternocleidomastoid muscle. In contrast to the raw $\text {sO}_2$ values, which are heavily affected by spectral coloring, the normalized TMR values are indicating reoxygenation of the lymph nodes in line with H1.

### Photoacoustic imaging reveals changes in water content indicating xerostomia

**Fig. 6:**
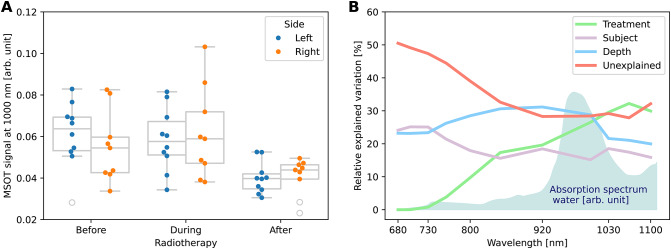
The photoacoustic signal of the submandibular gland at 1000 nm shows a strong correlation to the treatment phase (**A**). In line with this finding, the linear mixed model (LMM) analysis reveals an increase in explained photoacoustic signal variation for the treatment parameter (green) with longer wavelengths, where water becomes a primary absorber (**B**). Other LMM parameters like the individual (subject) and the depth of the structure stay constant or decrease with longer wavelengths like the unexplained (residual) signal variation. The box shows the interquartile range (IQR) with the median as center line. The whiskers extend to points that lie within 1.5 $\times$ IQR of the 1st or 3rd quantile. All data points are plotted on top, and color-coded by measurement side (left or right neck).

In addition to providing estimates of hemoglobin concentrations and hence $\text {sO}_2$, multispectral PAI promises to retrieve a diverse array of absorbers present in the near-infrared, enabling the investigation of a wide range of biological effects. Specifically, water emerges as a relevant chromophore for wavelengths around and above 1000 nm (absorption spectra Fig. 6B—background). We suggest linking water concentration to a prevalent RT side effect, xerostomia, characterized by patients suffering from dry mouth. The water content before and during the RT is approximately constant and comparable to the healthy volunteer group (Supplementary Fig. S6). Our measurements after RT indicate a decrease in water content visible in the PAI signal of the primary salivary gland (Fig. 6A), the submandibular gland, which is responsible for about 60% of saliva production in the unstimulated state^29^. This observation is supported by an LMM analysis for the PAI signal of the 11 measured wavelengths within the submandibular gland. The LMM analysis (Fig.6B) shows an increase in explained variation of the variable treatment with longer wavelengths. This goes alongside an increase of water absorption towards longer wavelengths and by declining inter-subject, depth, and particularly unexplained variability (residual variance).

## Discussion

This work presents the first in-human PAI study for monitoring the response of malignant lymph nodes in head and neck cancer patients to RT over time. Our study revealed radiation-induced changes in the surrounding tissue, including an increase in derived total hemoglobin in the sternocleidomastoid muscle, indicative of inflammation and revascularization, and a decrease in water content in salivary glands, suggesting xerostomia, confirming our hypotheses H2 and H3. Strikingly, PAI is capable of measuring molecular changes induced by RT in human tissue non-invasively. We also observed an increase in total hemoglobin within malignant lymph nodes, primarily comprising deoxygenated hemoglobin, resulting in a decline in $\text {sO}_2$. This decrease in $\text {sO}_2$ contradicts our initial hypothesis (H1) of observing reoxygenation within malignant lymph nodes resulting from RT treatment, as summarized in^21^. A digital twin model shed light on PAI-specific challenges in signal generation from malignant lymph nodes, particularly revealing low BVF as a factor associated with high prediction errors in $\text {sO}_2$. Our initial findings highlight the potential of PAI in directly monitoring tissue responses under RT at a biological scale, particularly inflammation, revascularization, and tissue malfunction, such as xerostomia, which has the potential to expand current clinical capabilities. To achieve these benefits PAI must overcome several barriers^30^. Notably, the limitations of photoacoustics, as described by Noltes et al.^31^, including acoustic reflections, penetration depth (fluence and spectral coloring), spectral cross-talk, and strong absorber coverage, are prominent in the data presented in this study. Additionally, reoxygenation effects predominantly occur in the hypoxic regions deep inside the lymph nodes, where fluence and coloring effects are most pronounced. As a treatment outcome, the nodes undergo shrinkage, posing challenges in matching individual nodes over time in particular for the last measurement. While a recent study shows the stability of PAI signal levels in healthy volunteers over 14 days^32^, anatomical changes due to the shrinkage of the lymph nodes, amongst others, limit the direct comparisons of PAI signals throughout the present study. Therefore, we also investigated relative measures such as the TMR and the explained variation with LMMs, which indicates high residual variance. Moreover, spectral coloring effects are particularly influential in shrinking nodes, as they often settle deeper in tissue post-shrinkage. The increase in tHb in surrounding tissue enhances the coloring effect. While the derived tumor-to-muscle ratio could potentially aid in identifying this phenomenon, this normalization should be interpreted cautiously to avoid confirmation bias and should be verified in future studies. A post hoc analysis of independently taken blood samples from the patients revealed a moderate correlation between tHb levels derived from the blood samples and PAI-derived tHb concentration within the target organs (Spearman correlation coefficient of 0.48 with p=0.08). Therefore, blood tHb levels may pose as a potential confounder, given the moderate correlation to the PAI signal, necessitating further investigation. Furthermore, malignant lymph nodes pose an aggravating factor due to their low BVF and high acoustic attenuation, resulting in particularly weak measurable photoacoustic signals. The importance of this effect was revealed through the presented digital twin model. However, it’s essential to note that the underlying simulations are constrained by a domain gap to real images^33^, especially since the effects of RT on surrounding tissue were not modeled.

Besides endeavors within the PAI community to tackle the quantification problem, identifying the most relevant feature(s) to address clinical problems is crucial. Therefore, we propose to further investigate tHb and relative measures as the TMR for monitoring RT response, since the interplay between oxy- and deoxyhemoglobin and current unmixing techniques might add more uncertainty in the low BVF regime of malignant lymph nodes (see digital twin analysis). Although oxygenation is highly relevant for assessing RT treatment response and tumor status, ambiguous measures for differences in $\text {sO}_2$ of malignant and benign thyroid nodules have been reported in recent PAI literature. Roll et al.^34^ observed significantly lower $\text {sO}_2$ in malignant than benign nodes and Noltes et al.^31^ “could not determine statistically relevant differences between benign and malignant thyroid nodules based on mean oxygen saturation in thyroid nodules”. Additionally, water content could be explored for assessing xerostomia. On the other hand, we see the importance of disentangling therapeutic effects from individual and PAI-specific uncertainties. This could be achieved by:

- Collecting larger datasets to understand heterogeneities in treatment response better.
- Implementing a meaningful normalization, such as the tumor-to-muscle ratio, to separate the therapeutic effect from individual variations.
- Identifying and addressing confounding factors that may influence PAI measurements.

Addressing these aspects may enhance the clinical utility of PAI for monitoring RT response and improving patient outcomes. In conclusion, the heterogeneity within the disease and the substantial signal variability arising from the current limitations of PAI highlight the challenges in monitoring RT treatment of malignant lymph nodes in head and neck cancer. Despite these obstacles, PAI shows promise as an early non-invasive assessment method for radiation response and could become a valuable tool in clinical practice, enhancing patient care and outcomes in head and neck cancer treatment.

## Methods

### Ethics

This study adheres to the Helsinki Declaration guidelines, was approved by the ethics committee of the Medical Faculty of Heidelberg University and was registered at clinicaltrials.gov (ID NCT04437030). All procedures were conducted with full informed consent from the participants.

### Study design, inclusion criteria, and participants

The PAI examinations were conducted on patients diagnosed with squamous cell carcinoma at various locations in the HN region, including the floor of the mouth, tonsil, and base of the tongue who received definitive radiotherapy without prior surgery. The patients had easily accessible lymph node metastases, primarily in levels I–III according to Gregoire’s classification^35^ (further tumor-related details see Tables 2 and 3 in the supplemental information). Originally, the study included 15 young healthy volunteers to train the physicians and test the measurement protocol, alongside 15 patients. Within the patient cohort, there were dropouts due to health conditions. Two patients were direct dropouts from the study, one patient died after radiotherapy, and one patient could no longer be examined at the third appointment due to declining health. Finally, the analysis included 11 complete patient datasets (three time points) and two incomplete patient datasets (two time points). 11 of the patients received moderately hypofractionated regimen (32 $\times$ 2.2 Gy with 70.4 Gy on tumor and 57.6 Gy on lymph node area)—one patient hypofractionated (due to health state) with 17 $\times$ 3 Gy (51 Gy), and one patient combined treatment with protons (57.6 Gy relative biological effectiveness (RBE) in weekly 5 $\times$ 1.8 Gy RBE single doses with simultaneous integrated boost (SIB) to the lymph nodes and the base of the tongue up to 64 Gy RBE and SIB to the tumor up to 70.4 Gy RBE). Also, 11 patients received concomitant systemic therapy (10$\times$ Cisplatin, 1$\times$ Cetuximab 5–6 Cycles). All patients underwent follow-up examination at 6–8 weeks after the end of therapy by CT or MRI, which allowed us to assess tumor response in another modality. Lymph node targets were CT- or MRI-graphically measured in two planes before radiotherapy and after the period described above. As a reference before radiotherapy, we used the lymph node metastases of interest which were seen in the radiotherapy planning CT scans. For treatment response the median follow-up period was 25 months (range 3–37). At the time of the last follow-up, 2 patients were dead for non-tumor related reasons. All patients showed initial response to radiotherapy. Two patients showed local progression at the primary tumor site after 15 and 19 months. There was one case of lymph nodal treatment failure after 12 months.

### Data acquisition and segmentation

**Fig. 7:**
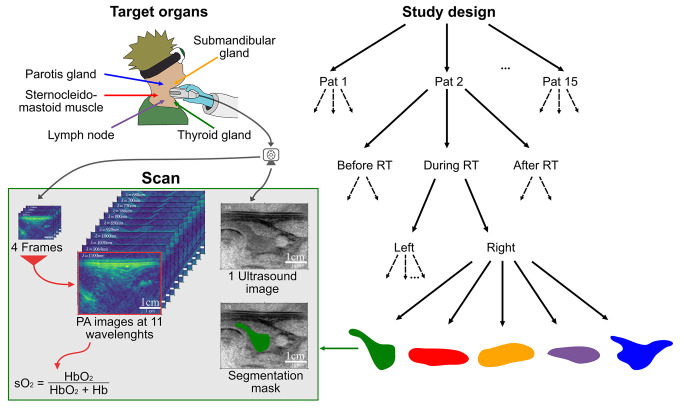
The study involved photoacoustic (PA) measurements of five target structures (sternocleidomastoid muscle, parotid gland, submandibular gland, thyroid gland, and if detectable benign or suspect malignant lymph nodes) measured before, during (after the 9th fraction), and 90 days after radiotherapy (RT). For each patient (Pat) and measurement, multispectral photoacoustic images, co-registered with an ultrasound image were taken from the left and right side. Clinical experts labeled the ultrasound images to generate semantic segmentation masks. Functional parameters like oxygen saturation ($\text {sO}_2$) were derived from the multispectral information using linear spectral unmixed concentrations of deoxygenated (Hb) and oxygenated ($\text {HbO}_2$) hemoglobin. This figure was created with Affinity Designer 2 (Version 2.5.3, https://affinity.serif.com/de/designer/).

For PA image generation, an MSOT Acuity Echo (iThera Medical GmbH, Munich) was used, which combines PAI with the possibility of co-registration by conventional ultrasound. The device uses a Nd:YAG laser with a tuning range of 660–1300 nm, a pulse peak energy of 30 mJ, a repetition rate of 25 Hz, and a pulse duration of 4–10 ns. The instrument is further equipped with an arc-shaped 1D detector array, with 256 elements and a center frequency of 4 MHz. Each study participant underwent measurements of five target organs before, during (after 9 RT fractions), and approximately 90 days after RT treatment on both the right and left side of the neck. Target organs included the sternocleidomastoid muscle, the parotid, the submandibular and the thyroid glands, and, if possible suspect malignant or benign lymph nodes. A graphical overview of the measuring procedure is displayed in Figure 7. Each scan (static snapshot) of every target organ comprised three technical replicates. For the healthy cohort, the three temporal measurements of the five target organs were performed on the same day consecutively. For acquisition eleven wavelengths (680, 700, 730, 760, 800, 850, 920, 1000, 1030, 1064, 1100 nm) were used and the image processing was conducted using the Simulation and Image Processing for Photonics and Acoustics (SIMPA) toolkit, involving correction for wavelength-dependent laser power, energy correction, bandpass filtering, and four frames average per wavelength, followed by delay and sum beamforming, envelope detection (Hilbert transform), and linear spectral unmixing for Hb and $\text {HbO}_2$ using five wavelengths (700–850 nm). Clinical experts performed the semantic annotation of co-registered US images with the medical imaging interaction toolkit (MITK)^36^ and the matching of the lymph nodes over time using CT/MRI images acquired in the standard clinical workflow.

### Linear mixed model analyis

The analysis of the explained variation was performed using the variance component form of the LMM. More specifically, we utilized separate linear mixed models for each wavelength to analyze explained variation and thus to evaluate the relevance (or importance) of factors contributing to changes in the observed absorbance values (Fig. 6). More precisely, linear mixed models were fitted for each wavelength separately, with fixed effects for the time/treatment, location (side), depth (of the structure), number of pixels in the region of interest (counts), smoking history, body mass index (BMI), and season of measurement (WS). Random effects were employed for the factor subject as well as the unexplained (residual) variation (Eq. 1). The proportion of explained variance was derived through the empirical decomposition of explained variation based on the variance components version of the mixed model^22^.

1 $$\begin{aligned} \begin{aligned} \text {absorbance}_{ij} = \alpha&+ \text {time}^\top _{ij} \cdot \beta _1 + \text {side}^\top _{ij} \cdot \beta _2 + \text {depth}^\top _{ij} \cdot \beta _3 + \text {counts}^\top _{ij} \cdot \beta _4 \\ &+ \text {smoking}^\top _{ij} \cdot \beta _5 + \text {BMI}^\top _{ij} \cdot \beta _6 + \text {WS}^\top _{ij} \cdot \beta _7 + \delta _i + \varepsilon _{ij}, \end{aligned} \end{aligned}$$

for repetition $j = 1, \ldots , n_i$ of patients $i=1, \ldots , 12$. The number of repetitions $n_i$ varied per patient. Here, $\alpha$ denotes a fixed intercept, and the $\beta _k$, $k = 1, \ldots , 6$ denote the effect sizes of the corresponding fixed effect. The random intercept $\delta _i\sim \mathcal {N}(0, \sigma _\delta ^2)$ describes subject specific variation. The residuals $\varepsilon _{ijk}\sim \mathcal {N}(0, \sigma _\varepsilon ^2)$ capture the residual or unexplained variation. Within the model, we assume that the random effects and the residuals are stochastically independent. Similarly, we used a single LMM to investigate the relevance of variables on the oxygenation of patients.

### Digital twin

The purpose of generating the digital twin model was to investigate the limitations of the study, in particular the oxygenation results contradicting H1, and the largely unexplained signal variability. For this purpose, all segmentation masks of malignant lymph nodes measured at all three time points were selected. All selected segmentation masks were refined for volume generation, by closing gaps and merging classes into dermis, subcutis, bulk tissue, and the lymph node class. Additionally, coupling media, membrane, and ultrasound gel were introduced. These refined masks were extrapolated outward to generate a 3D tissue model. Optical and acoustic properties were assigned to the tissue components using the SIMPA library based, among others, on work by Jacques et al.^37^ (see Tables 4–6 in the supplemental information). To mimic disease characteristics, different BVF and $\text {sO}_2$ values were assigned to every instance of the SIMPA lymph node object, specifically simulating three disease states: malignant (BVF 4 ± 2%, $\text {sO}_2$ 43 ± 15%), benign (BVF 14 ± 7%, $\text {sO}_2$ 73 ± 15%), and an artificial intermediate state (BVF 9 ± 4.5%, $\text {sO}_2$ 58 ± 15%), based on single reflectance spectroscopy measurements by Bugter et al.^24^. After volume generation, optical and acoustical simulation additive Gaussian noise was applied to the time series data using the SIMPA GaussianNoise adapter, to imitate a realistic noise level comparable to the real data (see full pipeline in Fig. 3). This was followed by image reconstruction using the same settings as for the real images.

### Usage of large language models

Large Language Models (LLMs) have been used to enhance the language of the manuscript. All model outputs have been thoroughly reviewed and verified for correctness by the authors.

## Supplementary Information

Supplementary Information.

## Data Availability

Individual de-identified participant data are not publicly available due to ethical restrictions. The data that supports the findings of this study are available upon reasonable request via email from the corresponding authors beginning with article publication and ending 36 months thereafter. The data usage is restricted to research purposes.
