# Supplementary material for: Photoacoustic imaging for monitoring radiotherapy treatment response in head and neck tumors
Source: Sci Rep. 2025 May 10;15:16344. doi: 10.1038/s41598-025-95137-0 (PMC12065922; doi:10.1038/s41598-025-95137-0)
Supplement: Supplementary file 1 — Supplementary Information. [file 41598_2025_95137_MOESM1_ESM.pdf]

Supplementary information: Photoacoustic imaging for monitoring radiotherapy treatment response in head and neck tumors

Niklas Holzwarth[1, 2, †, *], Zoe Rachel[3, †], Jan-Hinrich Nölke[1, 2], Melanie Schellenberg[1, 2, 4, 5], Lukas Bauer[3, 5], Nicholas Schreck[6, 7], Christoph J. Bender[1, 13], Kris K. Dreher[1, 8], Sebastian Regnery[3, 5], Katharina Weusthof[3, 5], Manuel Wiesenfarth[6], Annette Kopp-Schneider[6], Jürgen Debus[3, 5, 9], Alexander Seitel[1], Sebastian Adeberg[3, 10, 11, 12, ‡], Lena Maier-Hein[1, 2, 4, 5, 13, ‡, *], and Thomas Held[3, 5, ‡, *]

[1]Division of Intelligent Medical Systems (IMSY), German Cancer Research Center (DKFZ) Heidelberg, Germany
[2]Faculty of Mathematics and Computer Science, Heidelberg University, Germany
[3]Department of Radiation Oncology, Heidelberg University Hospital, Germany
[4]HIDSS4Health, Helmholtz Information and Data Science School for Health Heidelberg, Germany
[5]National Center for Tumor Diseases (NCT), NCT Heidelberg, a partnership between DKFZ and University Medical Center Heidelberg, Germany
[6]Biostatistics Division, German Cancer Research Center (DKFZ) Heidelberg, Germany
[7]Faculty of Liberal Arts and Sciences, Augsburg University of Applied Sciences, Germany
[8]Faculty of Physics and Astronomy, Heidelberg University, Germany
[9]Clinical cooperation unit radiotherapy, German Cancer Research Center (DKFZ) Heidelberg, Germany
[10]Department of Radiotherapy and Radiation Oncology, Marburg University Hospital, Germany
[11]Marburg Ion-Beam Therapy Center (MIT), Marburg University Hospital, Germany
[12]University Cancer Center (UCT), Frankfurt - Marburg, Germany
[13]Medical Faculty, Heidelberg University, Germany
[*]n.holzwarth@dkfz.de, l.maier-hein@dkfz.de, thomas.held@med.uni-heidelberg.de
[†]These authors contributed equally to this work.
[‡]These authors jointly supervised this work.

ABSTRACT

This supplementary information provides an additional evaluation of the paper data.

Time	Side	Depth	Counts	Smoking	BMI	WS	Subject-Lymph	Unexplained
32	3	1	-1	-3	-3	0	32	40

Table 1. We illustrate the variance decomposition for the endpoint oxygenation [%] derived from the linear mixed model (LMM) analysis. The variability in oxygen saturation (sO_2) in malignant patient lymph nodes is mainly unexplained, due to inter-target variability (subject-lymph) and due to time/treatment. Location (side), depth, pixel in the region of interest (counts), smoking history, body mass index (BMI), and season of measurement (WS) do not play a relevant role.

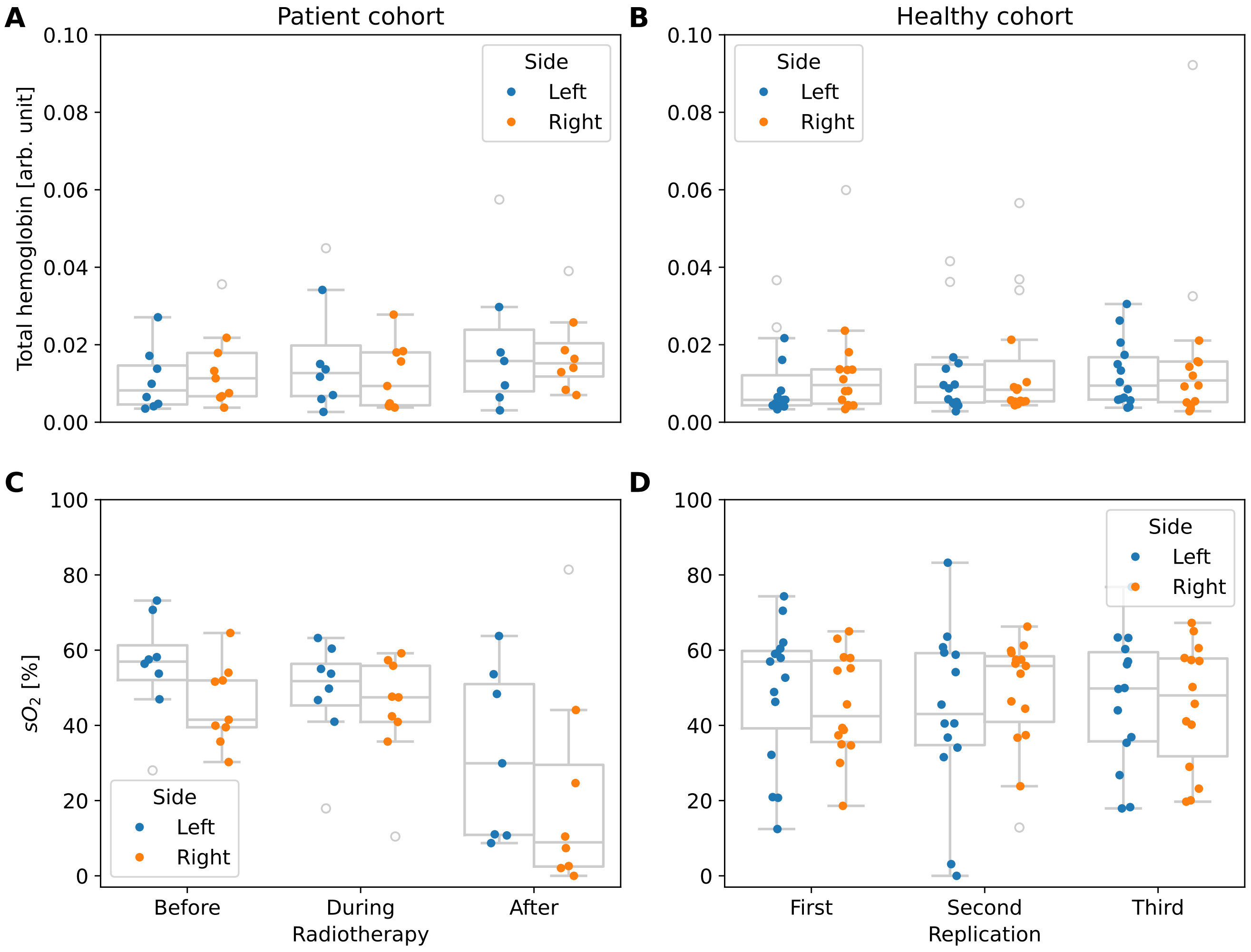

Figure 1. Biomarker distributions over time for total hemoglobin (A/B), as well as oxygen saturation (sO_2) (C/D), are shown for malignant patient (A/C) and benign healthy volunteer (no radiotherapy - B/D) lymph nodes. The box shows the interquartile range (IQR) with the median as center line. The whiskers extend to points that lie within 1.5 * IQR of the 1st or 3rd quantile. All data points are plotted on top, and color-coded by measurement side (left or right neck). Outliers are shown without color.

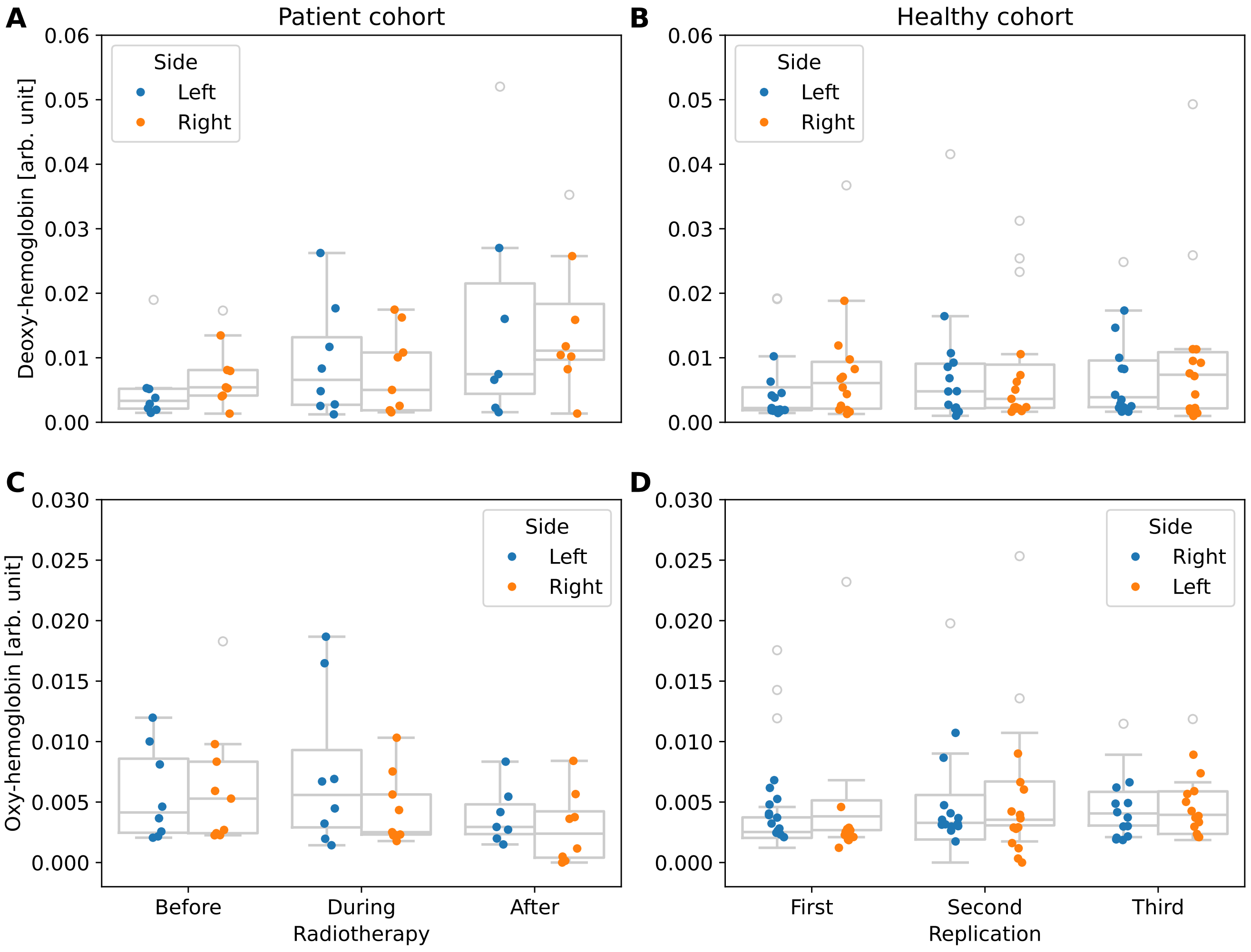

Figure 2. Biomarker distributions over time for deoxy- (A/B), oxy- (C/D) hemoglobin, are shown for malignant patient (A/C) and benign healthy volunteer (no radiotherapy - B/D) lymph nodes. The box shows the interquartile range (IQR) with the median as center line. The whiskers extend to points that lie within 1.5 * IQR of the 1st or 3rd quantile. All data points are plotted on top, and color-coded by measurement side (left or right neck). Outliers are shown without color.

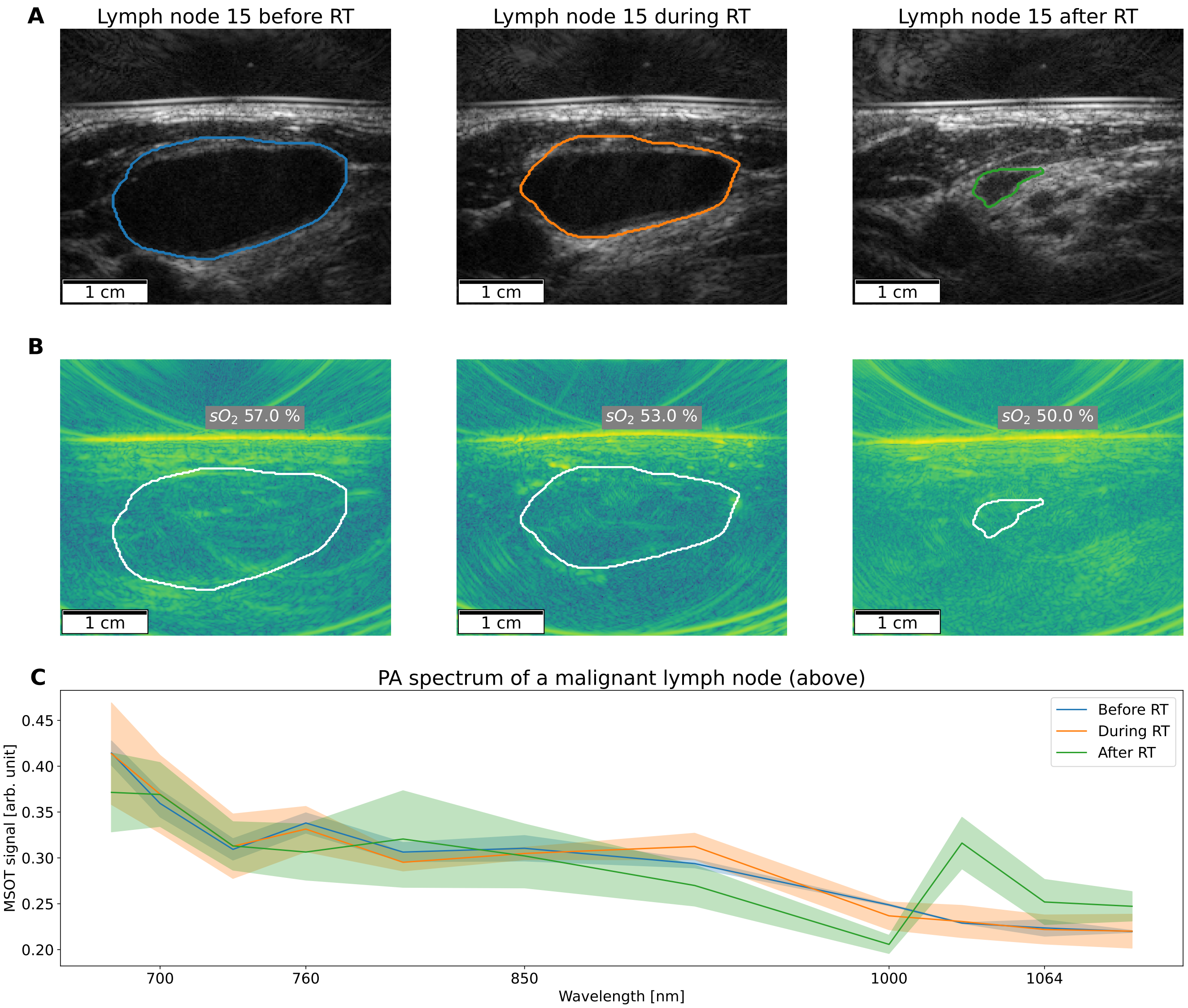

Figure 3. Example lymph node (ID 15) over time with corresponding ultrasound (US) images (A), photoacoustic (PA) images (B), and spectral information with standard deviation (C) derived from the region of interest (semantic segmentation of node as contour). Additionally, the derived oxygen saturation (sO_2) over the course of the radiotherapy (RT) is displayed in the PA image. The shrinking of the lymph node metastasis within three months can be attributed to a positive therapeutic response.

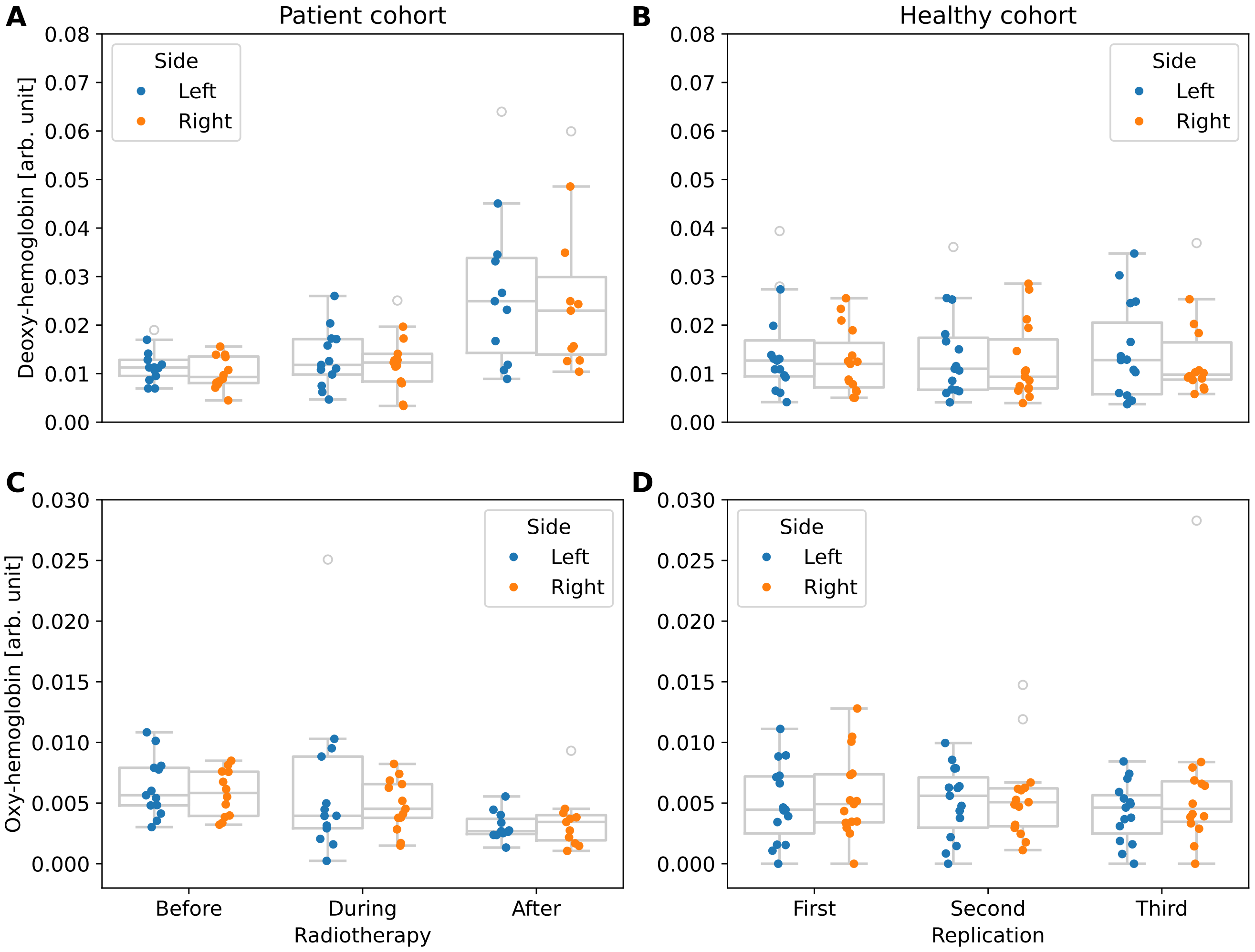

Figure 4. Strong increase in total hemoglobin within the sternocleidomastoid during radiotherapy (RT) is mainly caused by deoxyhemoglobin increase (A) and decrease in oxyhemoglobin (C) for patients undergoing RT treatment. For comparison, the healthy volunteer data (no RT) are displayed (B/D). The box shows the interquartile range (IQR) with the median as center line. The whiskers extend to points that lie within 1.5 * IQR of the 1st or 3rd quantile. All data points are plotted on top, and color-coded by measurement side (left or right neck). Outliers are shown without color.

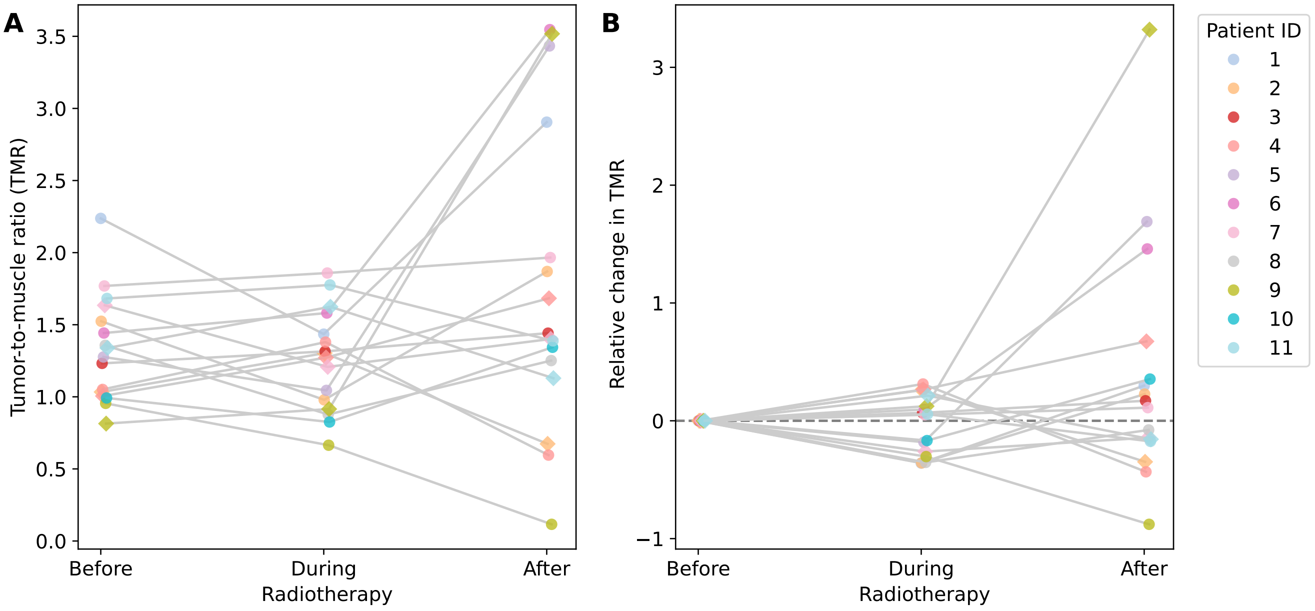

Figure 5. Tumor-to-muscle ratio (TMR) of oxygen saturation (sO_2) increases in suspect lymph nodes throughout radiotherapy (RT). This is shown for the overall TMR values (A) and relative to the first session (B), with each node color-coded by patient. For some patients, multiple nodes were measured, as indicated by their shape (1st node: circle; 2nd node: diamond shape).

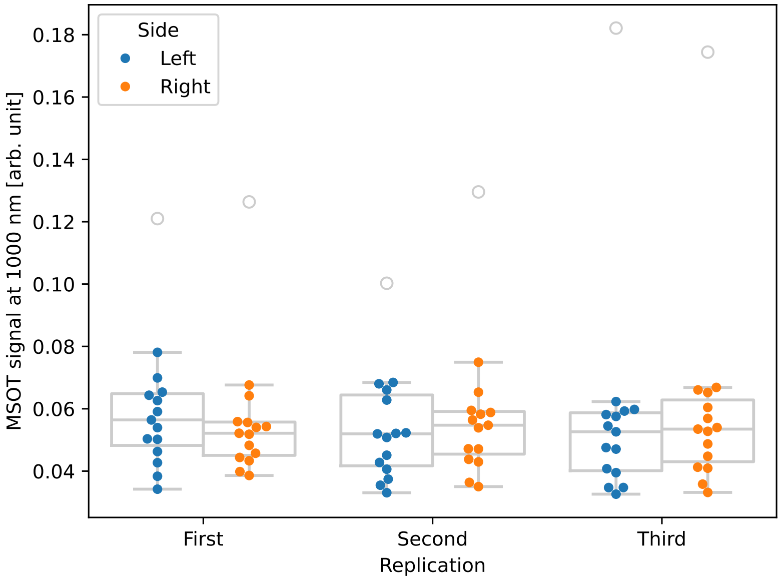

Figure 6. Photoacoustic signal of the submandibular gland at 1000 nm (near the water absorption peak) of healthy volunteers for all three repetitions. The box shows the interquartile range (IQR) with the median as center line. The whiskers extend to points that lie within 1.5 * IQR of the 1st or 3rd quantile. All data points are plotted on top, and color-coded by measurement side (left or right neck). Outliers are shown without color.

Lymph node ID	Location	Level	T	N	M	AJCC V8	Age	Sex
1	oropharynx	IIA	2	2	0	II	59	m
4/5	oropharynx	IIA/III	2	2	0	II	57	m
7	hypopharynx	III	3	3	0	IVB	73	m
8/10	oropharynx	IIA/III	4	3	1	IVB	66	m
11	oropharynx	V	3	2	0	II	66	m
15	oropharynx	IIB	4	2	0	III	64	m
16/19	hypopharynx	IIA/III	4	2	0	IVB	54	f
20	oral cavity	IIA	1	1	0	III	59	m
22/23	larynx	IIA/III	3	1	0	III	69	m
25	oropharynx	IIA	4	1	0	III	69	m
28/29	oropharynx	III/III	3	2	0	II	66	m

Table 2. Patient characteristics: The table gives an overview of the patient characteristics of the investigated tumors and the corresponding lymph nodes (IDs match Figure 2 - main paper). Histologically all tumors were classified as squamous cell carcinomas in the head and neck area. The locations of the primary tumors were heterogeneous with a dominance in the oropharyngeal space. The level describes the location of the lymph node metastases following the classification system of lymph node levels in head and neck cancers after Robbins et al.[1]. Tumor, lymph node, metastasis (TNM) classification and staging after the American Joint Committee on Cancer (AJCC) 8th edition[2] shows the extent of disease when patients were diagnosed and included in the study. The age at first diagnosis as well as the gender of the patient are also represented.

References

1. Robbins, K. T. *et al.* Neck dissection classification update: revisions proposed by the american head and neck society and the american academy of otolaryngology–head and neck surgery. *Arch. otolaryngology–head & neck surgery* **128**, 751–758 (2002).
2. Amin, M. B. *et al.* The eighth edition ajcc cancer staging manual: continuing to build a bridge from a population-based to a more "personalized" approach to cancer staging. *CA: a cancer journal for clinicians* **67**, 93–99 (2017).

Node ID	Size pre	Depth pre	Size during	Depth during	Size post	Depth post
1	0.9	2.5	1.1	0.6	0.9	2.3
4	0.7	0.9	0.7	1.0	1.2	0.9
5	0.8	1.4	1.3	1.3	0.9	1.4
7	1.5	0.9	1.4	0.4	0.9	1.6
8	1.5	1.5	1.3	1.7	1.0	1.4
10	1.5	0.6	1.8	0.6	1.2	1.2
11	1.0	0.6	1.2	1.0	1.1	0.6
15	1.7	0.9	1.6	0.8	0.4	1.8
16	1.2	0.8	0.6	2.4	0.5	0.6
19	0.7	1.3	0.8	1.1	0.7	1.0
20	1.8	0.7	1.1	0.6	0.8	0.9
22	1.2	1.2	1.0	1.1	0.6	1.4
23	0.7	1.7	0.8	1.8	1.0	0.8
25	1.4	1.3	1.3	1.8	1.1	1.6
28	1.0	1.1	0.8	1.6	0.8	1.2
29	1.1	0.4	0.8	0.6	1.0	1.2

Table 3. The table gives the lymph node size in cm^2 and depth in cm at each measurement before (pre), during (after the 9th fraction), and 90 days after the radiotherapy. The values were derived from the semantic segmentation masks.

LN	700 nm		730 nm		760 nm		800 nm		850 nm	
	min	max	min	max	min	max	min	max	min	max
μ_a	0.069	1.410	0.0590	1.052	0.083	1.510	0.052	1.180	0.074	1.380
μ_s	6.750	235.250	6.5007	226.250	6.260	217.930	5.970	207.780	5.640	196.390
g	0.994	0.999	0.9940	0.999	0.994	0.999	0.994	0.999	0.994	0.999

Fat	700 nm		730 nm		760 nm		800 nm		850 nm	
	min	max	min	max	min	max	min	max	min	max
μ_a	0.060	0.061	0.053	0.053	0.076	0.078	0.057	0.06	0.078	0.078
μ_s	58.410	60.630	57.035	59.440	55.750	58.330	54.190	56.96	52.410	55.390
g	0.968		0.968		0.968		0.968		0.968	

ST	700 nm		730 nm		760 nm		800 nm		850 nm	
	min	max	min	max	min	max	min	max	min	max
μ_a	0.060	0.255	0.052	0.198	0.076	0.284	0.057	0.224	0.078	0.270
μ_s	43.120	72.340	41.330	69.450	39.709	66.810	37.760	63.620	35.640	60.091
g	0.897	0.901	0.897	0.901	0.897	0.901	0.897	0.901	0.897	0.901

Table 4. Optical properties absorption coefficient (μ_a [cm^{-1}]), scattering coefficient (μ_s [cm^{-1}]), and anisotropy (g) for lymph node (LN), fat, and soft tissue (ST) used for the digital twin model.

Epidermis	700	730	760	800	850
μ_a [cm^{-1}]	3.0	2.0	2.0	1.0	1.0
μ_s [cm^{-1}]	52.0	49.0	47.0	44.0	41.0
g	0.804	0.813	0.823	0.835	0.849

Heavy water	700	730	760	800	850
μ_a [cm^{-1}]	0.001	0.001	0.001	0.001	0.001
μ_s [cm^{-1}]	1.0	1.0	1.0	1.0	1.0
g	1.0	1.0	1.0	1.0	1.0

Mediprene	700	730	760	800	850
μ_a [cm^{-1}]	0.017	0.017	0.017	0.017	0.017
μ_s [cm^{-1}]	0.147	0.147	0.147	0.147	0.147
g	0.9	0.9	0.9	0.9	0.9

US gel	700	730	760	800	850
μ_a [cm^{-1}]	0.006	0.018	0.025	0.02	0.043
μ_s [cm^{-1}]	1.0	1.0	1.0	1.0	1.0
g	1.0	1.0	1.0	1.0	1.0

Table 5. Optical properties absorption coefficient (μ_a), scattering coefficient (μ_s), and anisotropy (g) for the epidermis, heavy water (couplant), and ultrasound (US) gel used for the digital twin model.

	Density [kg/m^3]	SOS [m/s]	Ac. attenuation [dB/cm/MHz]
Epidermis	1109.0	1624.0	0.35
Fat (min)	975.5	1473.1	0.084
Fat (max)	982.2	1480.6	0.11
US gel	1000.0	1482.30	0.003
Heavy water	1107.0	1540.0	0.003
Mediprene	890.0	1583.0	0.277
Soft tissue (min)	1000.5	1501.2	0.063
Soft tissue (max)	1002.5	1502.7	0.069
Lymph node (min)	1032.8	1584.7	2.12
Lymph node (min)	1037.4	1587.1	2.83

Table 6. Acoustic properties density, speed of sound (SOS), and alpha coefficient/acoustic attenuation used for the digital twin model. US: ultrasound
